# Impaired cerebral autoregulation is associated with poststroke cognitive impairment

**DOI:** 10.1002/acn3.51075

**Published:** 2020-05-28

**Authors:** Nai‐Fang Chi, Han‐Hwa Hu, Lung Chan, Cheng‐Yen Wang, Shu‐Ping Chao, Li‐Kai Huang, Hsiao‐Lun Ku, Chaur‐Jong Hu

**Affiliations:** ^1^ Department of Neurology Shuang Ho Hospital Taipei Medical University New Taipei City Taiwan; ^2^ Department of Neurology School of Medicine College of Medicine Taipei Medical University Taipei Taiwan; ^3^ Department of Neurology Faculty of Medicine School of Medicine National Yang‐Ming University Taipei Taiwan; ^4^ Neurological Institute Taipei Veterans General Hospital Taipei Taiwan; ^5^ Department of Psychiatry School of Medicine College of Medicine Taipei Medical University Taipei Taiwan; ^6^ Deaparmtent of Psychiatry Shuang Ho Hospital Taipei Medical University New Taipei City Taiwan; ^7^ Brain and Consciousness Research Center Shuang Ho Hospital Taipei Medical University New Taipei City Taiwan; ^8^ Taipei Neuroscience Institute Taipei Medical University Taipei Taiwan; ^9^ The Ph.D. Program for Neural Regenerative Medicine College of Medical Science and Technology Taipei Medical University Taipei Taiwan

## Abstract

**Objective:**

To investigate whether dynamic cerebral autoregulation (CA) and neuroimaging characteristics are determinants of poststroke cognitive impairment (PSCI).

**Methods:**

Eighty patients within 7 days of acute ischemic stroke and 35 age‐ and sex‐matched controls were enrolled. In the patients with stroke, brain magnetic resonance imaging and dynamic CA were obtained at baseline, and dynamic CA was followed up at 3 months and 1 year. Montreal Cognitive Assessment (MoCA) was performed at 3 months and 1 year. Patients with a MoCA score <23 at 1 year were defined as having PSCI, and those with a MoCA score that decreased by 2 points or more between the 3‐month and 1‐year assessments were defined as having progressive cognitive decline.

**Results:**

In total, 65 patients completed the study and 16 developed PSCI. The patients with PSCI exhibited poorer results for all cognitive domains than did those without PSCI. The patients with PSCI also had poorer CA (lower phase shift between cerebral blood flow and blood pressure waveforms in the very low frequency band) compared with that of the patients without PSCI and controls at baseline and 1 year. CA was not different between the patients without PSCI and controls. In the multivariate analysis, low education level, lobar microbleeds, and impaired CA (very low frequency phase shift [≤46*°*] within 7 days of stroke), were independently associated with PSCI. In addition, impaired CA was associated with progressive cognitive decline.

**Interpretation:**

Low education level, lobar microbleeds, and impaired CA are involved in the pathogenesis of PSCI.

## Introduction

Cerebral autoregulation (CA) is the mechanism that minimizes changes in cerebral blood flow (CBF) during blood pressure fluctuations. Impaired CA results in unstable CBF and is detrimental to the outcome of neurological diseases, including subarachnoid hemorrhage, traumatic brain injury, and ischemic stroke.^1^, ^2^, ^3^, ^4^, ^5^, ^6^ Moreover, impaired CA is associated with neurodegenerative pathology, including cerebral amyloid deposition and white matter hyperintensities (WMHs).^7^ Therefore, CA may be a biomarker of both cerebrovascular and neurodegenerative diseases.

Poststroke cognitive impairment (PSCI) can hinder activities of daily living, decrease quality of life, and increase the healthcare burden.^8^ PSCI may occur immediately or months after a stroke. Early‐onset PSCI is caused by severe cerebral tissue loss or a lesion on the cognition‐related network, whereas the mechanisms of late‐onset PSCI are largely unclear. The prevalence of mild PSCI can be up to 52% 6 months after a stroke,^9^ but cognitive function may not be thoroughly assessed in all patients with stroke. Therefore, PSCI is a common but overlooked sequela of stroke. Because CA is impaired in patients with cerebrovascular or neurodegenerative diseases, impaired CA is likely to be a risk factor for late‐onset PSCI. Some neuroimaging characteristics such as cerebral microbleeds or WMHs are known risk factors for cognitive impairment and are common in patients with stroke.^10^ However, the relationships between CA, neuroimaging characteristics, and PSCI are unclear.

Dynamic CA is an approach to CA measurement in which CBF and peripheral blood pressure (BP) are noninvasively monitored in the resting state;^11^ therefore, it is feasible for clinical practice. In the present study, we followed up the temporal change in cognitive function and dynamic CA for 1 year in patients with acute ischemic stroke to determine whether dynamic CA indices are associated with the occurrence of PSCI at 1 year. In addition, we investigated the association between patients' dynamic CA and neuroimaging characteristics, including the presence of cerebral microbleeds and WMHs.

## Methods

The deidentified data employed in the current study are available to qualified investigators upon reasonable request.

### Participants

**Figure 1:**
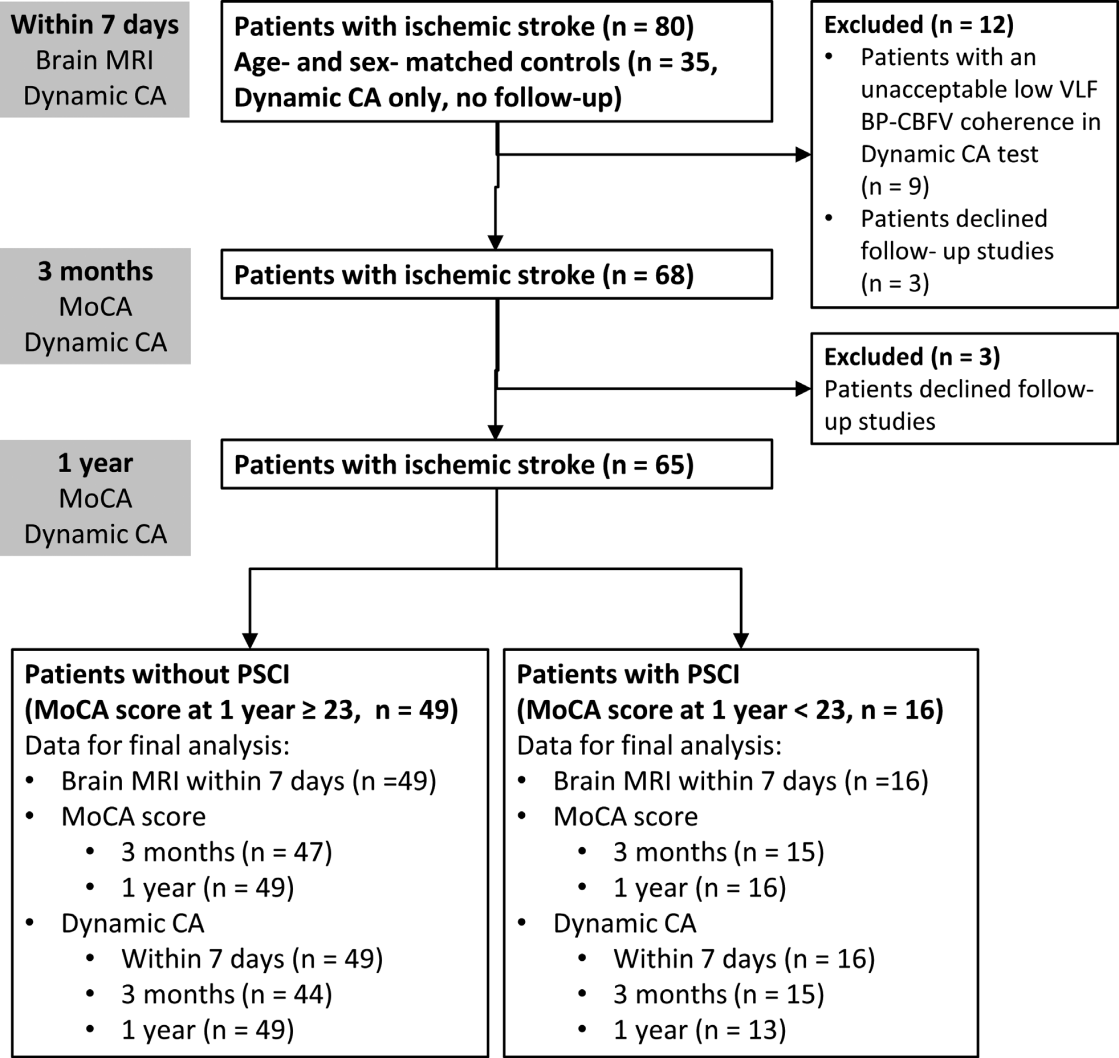
Flowchart of patient enrollment and the study protocol.

This study was approved by the Institutional Review Board of Taipei Medical University. Patients who were admitted to Taipei Medical University Shuang Ho Hospital within 7 days of acute ischemic stroke were consecutively screened for eligibility to participate in the current study. The exclusion criteria were as follows: (1) having a known cognitive impairment or a neurodegenerative disease that impairs daily activities before the stroke, (2) having a large cerebral infarct (greater than one third of the middle cerebral artery territory) or a strategic infarct (paramedian thalamus, medial frontal cortex, or hippocampus) that would cause early‐onset PSCI, (3) having a severe language or physical disability that impeded neuropsychological tests, (4) having atrial fibrillation (cognitive decline can be caused by a cardioembolism in the absence of clinical stroke^12^), and (5) no reliable dynamic CA result at the beginning of study. In total, 80 patients were recruited, and written informed consent was obtained from all participants or their legal guardians. Each patient was evaluated within 7 days of a stroke at admission and was followed up after 3 months and 1 year at the outpatient clinic. Six patients declined to participate in the follow‐up studies and were excluded. The data of 35 age‐ and sex‐matched healthy volunteers recruited in our past study were employed as control data.^13^ A flowchart of the patient enrollment and study protocol is provided (Fig. 1).

### Clinical characteristics, neurological and cognitive tests, and neuroimaging

The patients' stroke severity was evaluated using the National Institutes of Health Stroke Scale (NIHSS) at admission and at 1 year. Daily activity functional status was evaluated using the modified Rankin Scale (mRS) at 3 months and 1 year. Cognitive functions were evaluated using the Montreal Cognitive Assessment (MoCA) screening tool at 3 months and 1 year. A trained research assistant blinded to the results of patients' neuroimaging and dynamic CA conducted the neurological and cognitive assessments. Patients with a MoCA score <23 at 1 year were defined as having PSCI, and those with a MoCA score that decreased by 2 points or more between the 3‐month and 1‐year assessments were defined as having progressive cognitive decline.

The following brain magnetic resonance images (MRI) were obtained once at admission (GE Signa HDx 1.5T, General Electric Healthcare, Waukesha, WI, USA ): T1‐ and T2‐weighted images, T2 fluid‐attenuated inversion recovery (FLAIR) images, diffusion‐weighted images (DWIs), susceptibility‐weighted angiography (SWAN), and time‐of‐flight magnetic resonance angiogram (TOF MRA). Carotid Doppler ultrasonography and electrocardiogram were performed once at admission. The ischemic lesion volume was calculated using the DWI, and the severity of vascular stenosis was estimated using TOF MRA and carotid Doppler ultrasonography.^14^, ^15^ The distribution of cerebral microbleeds was determined using SWAN, and the severity of WMHs was evaluated using FLAIR images and the Fazekas scale.^16^ The images were interpreted by an experienced neurologist blinded to the patients' outcomes. Neurological and cognitive tests were conducted and neuroimaging was obtained in patients but not in controls.

### Dynamic cerebral autoregulation measurement and analysis

Dynamic CA was measured under spontaneous fluctuation in BP and CBF velocity (CBFV) during 5 min in a supine resting state. In brief, the CBFV of the extracranial internal carotid artery was recorded using a Doppler ultrasonography monitor (DWL MultiDop‐T, Compumedics DWL, Singen, Germany), and BP was recorded using a noninvasive BP monitor on the basis of finger plethysmography (Finometer Pro, Finapres Medical Systems, Enschede, The Netherlands), as in our previous studies.^1^, ^13^ The 5‐min CBFV and BP waveforms were recorded simultaneously, and a dynamic CA algorithm, namely transfer function analysis (TFA; the MATLAB code is available at http://www.car‐net.org/content/resources), was applied to calculate the phase shift, gain, and coherence between the BP and CBFV waveforms in the very low frequency (VLF, 0.02–0.07 Hz) and low frequency (LF, 0.07–0.20 Hz) bands.^11^ CA minimizes the changes in CBFV during spontaneous hemodynamic fluctuation; therefore, the changes in CBFV are smaller in amplitude and are restored to baseline faster than those in BP, which could be quantified as the gain and phase shift between BP and CBFV waveforms by using TFA. In patients with impaired CA, the gain is larger and phase shift is smaller than those in patients with normal CA.^11^, ^17^ In the current study, dynamic CA was tested within 7 days of stroke at admission, and the test was repeated at 3 months and 1 year at the outpatient clinic. In controls, dynamic CA was tested once. In total, 9 of the 80 patients were excluded after the first dynamic CA test because they had unacceptably low VLF coherence (<0.34 for a 5‐min recording)^11^ between BP and CBFV on the ipsilesional side. We did not have MoCA and dynamic CA results at 3 months for 2 of the 49 patients without PSCI and 1 of the 16 patients with PSCI because our research assistant could not reach the patients by telephone after they were discharged. Contact was established with these three patients when they returned to the outpatient clinic; therefore, they were able to complete the 1‐year follow‐up. In addition, we did not obtain dynamic CA data at 1 year for 3 of the 16 patients with PSCI because they were unwilling to undergo a dynamic CA test after finishing the MoCA. In total, 65 patients with complete data—namely brain MRI, first dynamic CA result, and MoCA at 1 year—were included in the final analysis (Fig. 1).

### Statistical analysis

Data normality was determined using the Shapiro–Wilk test. Normally distributed data are expressed as means ± standard deviations, whereas nonnormally distributed data are expressed as medians with interquartile ranges. Clinical characteristics, neuroimaging characteristics, and dynamic CA indices were compared between the patients with PSCI, the patients without PSCI, and controls by using the Kruskal–Wallis test or chi square test with post hoc analysis, as applicable. The average value of the bilateral sides of each dynamic CA index in the controls was compared with the ipsilesional side of each dynamic CA index in the patients. The patients' dynamic CA indices and MoCA scores were compared between different visits by using generalized estimating equations. The patients' dynamic CA indices were compared between bilateral sides by using the Wilcoxon signed‐rank test. Univariate logistic regression was conducted to determine the odds ratio of developing PSCI related to the clinical characteristics, neuroimaging characteristics, and dynamic CA indices. Receiver operating characteristic analysis with Youden's J statistic was used to test the sensitivity and specificity and determine the optimal cut‐off value of dynamic CA indices for identifying patients more likely to develop PSCI. Multivariate logistic regression using automatic forward variable selection was conducted to construct a model including significant variables associated with PSCI (variables were eligible for inclusion in the model if *P* < 0.10). A *P* value of <0.05 was considered statistically significant. The data were analyzed using MedCalc Statistical Software v19 (MedCalc Software bvba, Ostend, Belgium) and PASW Statistics v18 (SPSS Inc. Chicago, IL, USA).

## Results

The clinical characteristics of the patients with PSCI (*n* = 16), the patients without PSCI (*n* = 49), and controls (*n* = 35) were summarized in Table 1. Age and sex were not different between the patients (*n* = 86) and controls. Most patients had mild stroke severity (median NIHSS score = 4) and small vessel disease, which was consistent with our study inclusion and exclusion criteria. The patients with PSCI had more advanced age, lower education level, and a higher lobar microbleed burden than did those without PSCI. The prevalence of vascular risk factors (hypertension, diabetes mellitus, hyperlipidemia, and stenosis of internal carotid or middle cerebral artery), stroke severity (baseline NIHSS score and DWI lesion volume), stroke etiologies, WMH severity (Fazekas scale score), and the burden of deep or infratentorial microbleeds were not different between the patients with and without PSCI. In both of these groups, the NIHSS scores at 1 year were significantly lower than those obtained within 7 days, suggesting that the neurological deficits improved regardless of the onset of PSCI.

**Table 1:** Clinical and neuroimaging characteristics of the participants

	Controls (*n* = 35)	PSCI (−) (*n* = 49)	PSCI (+) (*n* = 16)	*P* value
Age, median (IQR)	59 (55–64)	56 (49–62)	64 (51–69)^1^	0.048*
Male gender	26 (74%)	35 (71%)	13 (81%)	0.738
Education level		12 (9–16) years	9 (6–12) years	0.005*
Hypertension	12 (34%)	38 (78%)	9 (56%)	<0.001*
Diabetes mellitus	6 (17%)	16 (33%)	6 (38%)	0.193
Hemoglobin A1c within 7 days, median (IQR)		5.9 (5.6–6.5)%	5.7 (5.5–7.9)%	0.512
Hyperlipidemia	12 (34%)	35 (71%)^2^	11 (69%)^2^	0.002*
NIHSS score within 7 days, median (IQR)		4 (2–5)	4 (2–6)	0.988
NIHSS score at 1 year, median (IQR)		0 (0–1)^3^	1 (1–4)^3^	0.006*
Stroke etiology				0.687
Large artery atherosclerosis		13 (27%)	3 (19%)	
Small vessel disease		31 (63%)	12 (75%)	
Undetermined etiology		5 (10%)	1 (6%)	
DWI lesion volume, median (IQR)		0.7 (0.3–2.8) cc	0.4 (0.3–3.3) cc	0.653
DWI lesion on the left side		23 (47%)	11 (69%)	0.132
Fazekas scale score, periventricular + deep white matter, median (IQR)		1 (1–2)	2 (1–4)	0.142
Presence of cerebral microbleeds				
Lobar		11 (22%)	8 (50%)	0.037*
<4 microbleeds		6 (12%)	6 (38%)	
≥4 microbleeds		5 (10%)	2 (13%）	
Deep		11 (22%)	4 (25%)	0.835
<4 microbleeds		6 (12%)	4 (25%)	
≥4 microbleeds		5 (10%)	0 (0%)	
Infratentorial		6 (12%)	2 (13%)	0.979
<4 microbleeds		4 (8%)	2 (13%)	
≥4 microbleeds		2 (4%)	0 (0%)	
ICA or MCA stenosis ≥ 70% on either side		5 (10%)	0 (0%)	0.187

DWI, diffusion‐weighted image; ICA, internal carotid artery; IQR, interquartile range; MCA, middle cerebral artery; MoCA, Montreal Cognitive Assessment; mRS, modified Rankin Scale; NIHSS, National Institutes of Health Stroke Scale; PSCI, poststroke cognitive impairment.

^1^ Different from PSCI(−).

^2^ Different from controls.

^3^ Different from the result within 7 days.

* *P* < 0.05.

In the patients with PSCI, all MoCA subdomain scores including visuospatial and executive function, naming, attention, language, abstraction, delayed recall, orientation, and total scores were significantly lower than those in the patients without PSCI at both 3 months and 1 year (Table 2). In the patients with PSCI, all MoCA subdomain scores were not significantly different between 3 months and 1 year, although deterioration in visuospatial and executive function, attention, and delayed recall was observed between 3 months and 1 year. The patients without PSCI had a significantly higher score for abstraction and total score at 1 year compared with those at 3 months; the remaining MoCA subdomain scores were not different between these two time points. In the patients with PSCI, the prevalence of progressive cognitive decline was significantly higher than that in the patients without PSCI (40% vs. 6%, *P* = 0.001). In the patients with progressive cognitive decline, delayed recall was the only cognitive domain in which they deteriorated between 3 months and 1 year (median score decreased from 3 to 0; Table 3).

**Table 2:** MoCA scores of the patients with and without PSCI.

Cognitive domain	Scores, median (IQR)		*P* value
	PSCI (−) *n* = 47 at 3 months, *n* = 49 at 1 year	PSCI (+) *n* = 15 at 3 months, *n* = 16 at 1 year	
Visuospatial and executive function (0–5)			
3 months	4 (4–5)	3 (0–4)	<0.001*
1 year	4 (4–5)	2 (0–4)	<0.001*
Naming (0–3)			
3 months	3 (3–3)	3 (1–3)	<0.001*
1 year	3 (3–3)	3 (2–3)	<0.001*
Attention (0–6)			
3 months	6 (5–6)	5 (3–6)	0.015*
1 year	6 (6–6)	4 (3–6)	<0.001*
Language (0–3)			
3 months	2 (2–3)	1 (0–2)	<0.001*
1 year	2 (2–3)	1 (0–2)	<0.001*
Abstraction (0–2)			
3 months	1 (1‐ 2)	1 (0–1)	0.012*
1 year	2 (1–2)**	1 (0–1)	<0.001*
Delayed recall (0–5)			
3 months	4 (3–5)	0 (0–3)	<0.001*
1 year	4 (3–5)	0 (0–2)	<0.001*
Orientation (0–6)			
3 months	6 (6–6)	4 (2–6)	<0.001*
1 year	6 (6–6)	5 (3–6)	<0.001*
Total score (0–30)			
3 months	26 (24–28)	18 (10–23)	<0.001*
1 year	27 (26–28)**	18 (12–22)	<0.001*
Total score change from 3 months to 1 year	+1 (−1 to +2)	0 (−4 to +3)	0.179
Progressive cognitive decline (total score decreased 2 or more from 3 months to 1 year)	3 (6%)	6 (40%)	0.001*

IQR, interquartile range; MoCA, Montreal Cognitive Assessment; PSCI, poststroke cognitive impairment.

* *P* < 0.05.

** *P* < 0.05 in comparison with 3 months.

**Table 3:** Cognitive domains in MoCA of the patients with progressive cognitive decline after stroke (*n* = 9).

Cognitive domain	Scores at 3 months, median (IQR)	Scores at 1 year, median (IQR)	*P* value
Visuospatial and executive function (0–5)	3 (3–4)	4 (1–4)	0.438
Naming (0–3)	3 (3–3)	3 (2–3)	1.000
Attention (0–6)	6 (6–6)	5 (4–6)	0.094
Language (0–3)	2 (1–3)	2 (1–3)	1.000
Abstraction (0–2)	1 (1–2)	1 (0–2)	0.813
Delayed recall (0–5)	3 (2–5)	0 (0–3)	0.016*
Orientation (0–6)	6 (5–6)	6 (5–6)	0.188
Total score (0–30)	26 (22–28)	22 (18–25）	0.004*

Progressive cognitive decline was defined as a decrease of 2 points or more in the MoCA score at 1 year compared with that at 3 months. IQR, interquartile range; MoCA, Montreal Cognitive Assessment.

* *P* < 0.05.

**Figure 2:**
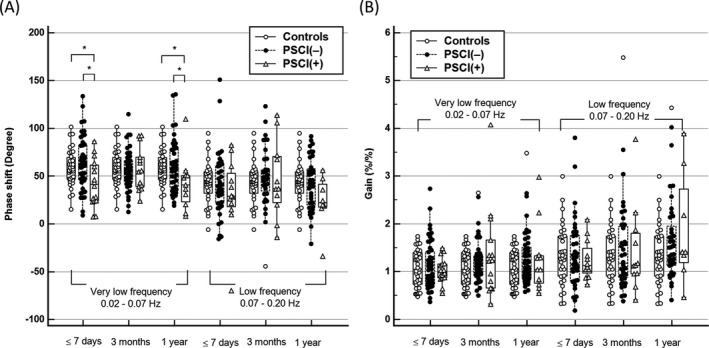
Comparison of the ipsilesional dynamic cerebral autoregulation indices between groups: (A) phase shift and (B) gain. *Significant difference in the post hoc analysis of the Kruskal–Wallis test.

A dynamic CA comparison was performed of the patients with PSCI, the patients without PSCI, and controls (Fig. 2). The VLF phase shift of the patients with PSCI was significantly lower than that of the patients without PSCI and controls within 7 days and at 1 year. No difference in VLF phase shift was observed between the patients without PSCI and controls nor any difference in the LF phase shift between the three groups (Fig. 2A). The gain was also not significantly different between the three groups for both the VLF and LF bands (Fig. 2B).

The results of univariate logistic regression of clinical and neuroimaging characteristics in relation to PSCI were summarized in Table 4. Education level and presence of lobar microbleeds were significant predictors of PSCI, whereas age and Fazekas scale score were predictors with borderline significance. The other clinical and neuroimaging characteristics including sex, hypertension, diabetes mellitus, hemoglobin A1c level, hyperlipidemia, NIHSS score within 7 days, DWI lesion volume and side, and presence of deep or infratentorial microbleeds were not predictors of PSCI. The results of univariate logistic regression for hemodynamics and CA were summarized in Table 5. Mean BP and mean CBFV (5‐min average of BP and CBFV waveform, respectively) at all visits were not predictors of PSCI. Ipsilesional VLF phase shift within 7 days and bilateral VLF phase shift at 1 year were significant predictors of PSCI, whereas contralesional VLF phase shift within 7 days was a predictor of PSCI with borderline significance. VLF phase shift at 3 months and VLF gain at all visits were not predictors of PSCI. In addition, LF phase shift and gain were not predictors of PSCI at any visit. The optimal value of the ipsilesional VLF phase shift within 7 days in predicting PSCI was ≤ 46°; therefore, we defined VLF phase shift ≤ 46° as “impaired CA” and used this criterion to evaluate CA during subsequent visits as well as on the contralesional side. Impaired CA on either the ipsilesional or contralesional side within 7 days was a predictor of PSCI and so was impaired CA on the contralesional side at 1 year, whereas impaired CA at 3 months was not a predictor of PSCI.

**Table 4:** Univariate logistic regression analyses: clinical and neuroimaging characteristics as predictors of PSCI.

Characteristics (*n* = 65)	Odds ratio (95% CI)	*P* value
Age	1.06 (0.99–1.13)	0.079
Male gender	1.73 (0.43–7.03)	0.441
Education level (year)	0.76 (0.63–0.91)	0.003*
Hypertension	0.37 (0.11–1.23)	0.105
Diabetes mellitus	1.24 (0.38–4.01)	0.722
Hemoglobin A1c (%) within 7 days	1.05 (0.81–1.35)	0.724
Hyperlipidemia	0.88 (0.26–3.00)	0.838
NIHSS score within 7 days	1.03 (0.85–1.24)	0.785
DWI lesion volume (cm^3^)	1.01 (0.96–1.06)	0.813
DWI lesion on the left side	2.49 (0.75–8.23)	0.136
Fazekas scale score (periventricular + deep white matter)	1.43 (0.99–2.09)	0.059
Presence of cerebral microbleeds		
Lobar	3.45 (1.05–11.33)	0.041*
Deep	1.15 (0.31–4.29)	0.833
Infratentorial	0.81 (0.20–3.25)	0.762

DWI, diffusion‐weighted image; MoCA, Montreal Cognitive Assessment; mRS, modified Rankin Scale; NIHSS, National Institutes of Health Stroke Scale; PSCI, poststroke cognitive impairment.

* *P* < 0.05.

**Table 5:** Univariate logistic regression analyses: hemodynamics and CA as predictors of PSCI.

Hemodynamics parameters	*N*	Within 7 days		*N*	3 months		*N*	1 year	
		Odds ratio (95% CI)	*P* value		Odds ratio (95% CI)	*P* value		Odds ratio (95% CI)	*P* value
Mean blood pressure (mmHg)	65	1.00 (0.97–1.03)	0.955	59	0.97 (0.94–1.01)	0.181	62	0.99 (0.96–1.03)	0.632
Mean cerebral blood flow velocity (cm/s)									
Ipsilesional	65	0.99 (0.94–1.04)	0.629	59	1.01 (0.96–1.06)	0.775	62	1.00 (0.92–1.07)	0.930
Contralesional	65	0.99 (0.94–1.04)	0.681	59	0.96 (0.91–1.02)	0.157	62	0.96 (0.91–1.03)	0.251
Cerebral autoregulation									
VLF Phase shift (degree)									
Ipsilesional	65	0.98 (0.95–1.00)	0.031*	58	0.99 (0.97‐ 1.02)	0.655	60	0.97 (0.94–1.00)	0.048*
Contralesional	59	0.98 (0.95–1.00)	0.051	56	0.99 (0.96–1.02)	0.491	58	0.97 (0.93–1.00)	0.045*
VLF Gain (%/%)									
Ipsilesional	65	0.62 (0.16–2.40)	0.489	58	1.53 (0.61–3.86)	0.363	60	0.99 (0.33–3.01)	0.986
Contralesional	59	3.24 (0.79–13.26)	0.102	56	1.59 (0.51–4.89)	0.422	58	0.78 (0.20–3.00)	0.712
LF Phase shift (degree)									
Ipsilesional	53	0.99 (0.97–1.01)	0.307	52	1.00 (0.98–1.01)	0.638	48	0.97 (0.94–1.00)	0.086
Contralesional	51	0.98 (0.97–1.00)	0.100	53	1.00 (0.98–1.02)	0.843	51	1.00 (0.98–1.01)	0.525
LF Gain (%/%)									
Ipsilesional	53	0.81 (0.28–2.31)	0.689	52	0.97 (0.48–1.97)	0.941	48	1.26 (0.58–2.73)	0.562
Contralesional	51	1.72 (0.92–3.22)	0.090	53	1.62 (0.68–3.85)	0.272	51	0.99 (0.51–1.92)	0.967
Impaired cerebral autoregulation (VLF Phase shift ≤ 46°)^1^									
Ipsilesional	65	5.14 (1.54–17.12)	0.008*	58	2.55 (0.75–8.66)	0.135	60	2.48 (0.66–9.34)	0.181
Contralesional	59	3.66 (1.02–13.18)	0.047*	56	1.44 (0.40–5.26)	0.579	58	6.97 (1.60–30.42)	0.010*

LF, low frequency (0.07–0.20 Hz); VLF, very low frequency (0.02–0.07 Hz); PSCI, poststroke cognitive impairment.

^1^ The optimal value of ipsilesional VLF phase shift within 7 days in identifying PSCI was ≤46°, with sensitivity = 62.5%, specificity = 75.5%, and area under the receiver operating characteristics curve = 0.714 (*P* = 0.006).

* *P* < 0.05.

The results of multivariate logistic regression were summarized in Table 6. We entered the clinical and neuroimaging characteristics as well as the ipsilesional VLF phase shift within 7 days into the regression model. By using the automatic forward variable selection method, the education level, presence of lobar microbleeds, and VLF phase shift were selected into the model, implying that they were independent predictors of PSCI. When we entered “impaired CA within 7 days (ipsilesional VLF phase shift ≤ 46°)” into the regression model instead of “VLF phase shift within 7 days,” educational level, presence of lobar microbleeds, and impaired CA within 7 days remained independent predictors of PSCI (odds ratios of the presence of lobar microbleeds and impaired CA were 6.23 [*P* = 0.020] and 5.77 [*P* = 0.020] respectively).

**Table 6:** Multivariate logistic regression analyses of PSCI.

Characteristics (*n* = 65)	Odds ratio^1^ (95% CI)	*P* value	Odds ratio^2^ (95% CI)	*P* value
Education level (year)	0.72 (0.57–0.90)	0.004*	0.76 (0.62–0.93)	0.008*
Presence of lobar microbleeds	8.50 (1.63–44.31)	0.011*	6.23 (1.34–28.94)	0.020*
Ipsilesional cerebral autoregulation				
VLF Phase shift within 7 days (degree)	0.96 (0.93–0.99)	0.012*		
Impaired cerebral autoregulation within 7 days (VLF Phase shift ≤ 46°)			5.77 (1.31–25.41)	0.020*

PSCI, poststroke cognitive impairment; VLF, very low frequency (0.02–0.07 Hz).

^1^ Model including education level, presence of lobar microbleeds, and VLF phase shift within 7 days.

^2^ Model including education level, presence of lobar microbleeds, and impaired cerebral autoregulation within 7 days.

* *P* < 0.05.

The comparison of systemic and cerebral hemodynamic parameters between different visits is provided in Table S1. Phase and gain were not significantly different between all visits for either the VLF or LF band in the patients with and without PSCI. Mean CBFV, phase shift, and gain were not significantly different between bilateral sides at the same visit in the patients with and without PSCI. Moreover, mean BP and mean CBFV were not significantly different between visits in both the patients with and without PSCI, nor were they significantly different between these patients at the same visit.

Impaired CA was not associated with the severity of initial neurological deficit (NIHSS score), severity of WMHs (Fazekas scale score), or presence of microbleeds (Table S2). In addition, progressive cognitive decline was associated with impaired CA but not with clinical characteristics, the severity of WMHs, or the presence of microbleeds (Table S3).

## Discussion

In the current study, we followed up 65 patients with ischemic stroke for 1 year to investigate the risk factors of PSCI. Low education level, presence of lobar microbleeds, and impaired CA were identified as independent risk factors of PSCI. Patients with PSCI exhibited poorer results in all cognitive domains compared with the patients without PSCI. Although the patients with PSCI had higher incidence of progressive cognitive decline than did those without PSCI, more than half of the patients with PSCI exhibited stability or improvement in cognitive function. This finding suggests that the pathogenesis of PSCI involves poor cognitive function before or after stroke in most patients and progressive cognitive decline in some patients. Impaired CA was a risk factor not only of PSCI but also of progressive cognitive decline. In addition, impaired CA was not associated with the severity of initial neurological deficit, WMHs, or the presence of lobar microbleeds. Therefore, impaired CA is an important risk factor of PSCI and is likely not the consequence of a pre‐existing brain lesion or acute minor stroke.

In related studies, old age, low education level, diabetes, and atrial fibrillation have been found to increase the risk of PSCI.^18^, ^19^ However, these factors also increase the risk of prestroke cognitive impairment^18^ and have been associated with Alzheimer disease (AD).^20^, ^21^ Therefore, cognitive impairment and stroke have the same risk factors, and patients with PSCI might actually have had subclinical prestroke cognitive impairment despite reporting normal premorbid cognitive function. Nevertheless, the occurrence of a stroke accelerates cognitive decline, and the effect is stronger in patients who are older and have experienced cardioembolic stroke.^19^ In the current study, although education level was lower in the patients with PSCI than in the patients without PSCI, education level was not different between the patients with and without progressive cognitive decline (Table S3). Therefore, low education level may reflect poor baseline cognitive function but is not a risk factor of progressive cognitive decline.

Neuroimaging characteristics including strategic stroke, stroke lesion volume, total brain tissue volume, temporal lobe atrophy, WMHs, and microbleeds were reported to be associated with PSCI.^22^ In the current study, stroke lesion volume and WMHs were not associated with PSCI, and lobar microbleeds, but not deep or infratentorial types, were associated with PSCI. This discrepancy might be explained by the patient characteristics in the current study. The patients had uniformly low burden of pathological neuroimaging characteristics, including small infarction, mild WMHs, and few microbleeds (Table 1). Therefore, the neuroimaging characteristics may not have affected cognitive function to a great extent in the current study. Nevertheless, the presence of lobar microbleeds, even those that were not severe, was associated with PSCI in the current study. This could be because lobar microbleeds indicate the presence of subclinical neurodegenerative pathomechanisms such as AD‐related cerebral amyloid angiopathy.^23^, ^24^ The prevalence of cerebral amyloid pathology, detected using positron emission tomography (PET), was approximately 10% in patients with ischemic stroke^25^ and approximately 20% in patients with PSCI.^26^ The prevalence of a positive amyloid PET result in patients with PSCI was not higher than that in patients without PSCI.^25^ Therefore, both preexisting amyloid pathology and other factors contribute to the development of PSCI.

A novel finding that emerged from the current study is that impaired CA is associated with PSCI. Impaired CA results in unstable CBF and secondary injury after a stroke,^27^ and impaired CA is associated with reduced functional connectivity in cognition‐related networks.^28^ In related studies, impaired CA was associated with large lesion volume and elevated BP, but it also independently predicted poor functional recovery from stroke.^1^, ^3^, ^5^ In the current study, the VLF, but not the LF, phase shift was associated with PSCI. The clinical or physiological significance of different frequency bands is not yet well‐understood. Nevertheless, the literature suggests that neural network activity is associated with VLF spontaneous fluctuation, whereas vasomotion and sympathetic activity are associated with LF spontaneous fluctuation.^29^, ^30^ VLF, but not LF, phase shift has been reported to be associated with the functional outcomes of ischemic stroke and traumatic brain injury.^1^, ^31^ Therefore, VLF phase shift may reflect the functioning of the neurovascular unit, and impaired CA may be detrimental to poststroke cognitive function recovery through its negative influences on cognition‐related networks. Most patients enrolled in the current study did not have significant cerebrovascular stenosis. Although cerebrovascular stenosis results in impaired CA, patients without cerebrovascular stenosis may still exhibit impaired CA. Consistent with our results, studies have reported that patients with lacunar infarction had impaired CA in bilateral hemispheres.^32^, ^33^ The possible mechanism behind this phenomenon is neurovascular unit dysfunction in small vessel disease,^34^ which is consistent with the finding that VLF phase shift is the optimal CA index for predicting poststroke cognitive impairment. Patients with AD were found to have normal CA,^35^ which is consistent with the lack of association between CA and lobar microbleeds observed in the current study. Therefore, CA is likely independent of AD‐related pathology.

No dynamic CA indices changed significantly between all visits in the current study regardless of the onset of PSCI and improvement in NIHSS score. In a 6‐month longitudinal study, patients with lacunar infarction exhibited sustained impaired CA,^32^ which is consistent with the results of current study. This finding suggests that regular healthcare was unable to alter CA in patients with minor stroke despite some neurological improvement. Some therapeutic interventions, including antihypertensive medicines and remote ischemic preconditioning, can alter CA^36^, ^37^, ^38^ and thus may represent a novel strategy to prevent the onset of PSCI.

The current study had some limitations. First, although PSCI refers to cognitive impairment after a stroke, subclinical cognitive impairment might have been present before the stroke; however, all participants of the current study reported normal premorbid cognitive function. Second, patients with atrial fibrillation were excluded, and these patients might account for 10 to 15% of all patients with ischemic stroke.^39^, ^40^ Third, most patients had mild disease. Fourth, this study included relatively few participants from a single hospital, which may have affected the generalizability of the findings. Fifth, 9 of the 80 patients were excluded at the first visit because of unacceptably low VLF coherence of BP and CBFV in the TFA algorithm. A longer dynamic CA recording time may reduce the incidence of this problem.

In conclusion, the occurrence of PSCI is influenced by low education level, the presence of lobar microbleeds, and impaired CA. In addition, impaired CA is a risk factor of progressive cognitive decline. Treating impaired CA may be a novel strategy for preventing PSCI.

## Author Contribution

N‐F Chi, H‐H Hu and C‐J Hu: conceptualization and methodology. N‐F Chi, L Chan, S‐P Chao, L‐K Huang, H‐L Ku, and C‐J Hu: data acquisition. N‐F Chi, H‐H Hu, C‐Y Wang, H‐L Ku, and C‐J Hu: data analysis. N‐F Chi, H‐H Hu, and C‐J Hu: funding acquisition and supervision. N‐F Chi and C‐J Hu: manuscript drafting. All authors: manuscript review and editing.

## Funding Information

This study was supported by the Ministry of Science and Technology of Taiwan (MOST 106‐2314‐B‐038‐001, 107‐2314‐B‐038‐051, 108‐2314‐B‐038‐056, 108‐2314‐B‐038‐004, 108‐2314‐B‐038‐005, 108‐2314‐B‐075‐018‐MY2).

## Conflict of Interest

None.

## Supporting information


**Table S1.** Comparison of systemic and cerebral hemodynamic parameters between different visits.
**Table S2.** Clinical and neuroimaging characteristics of the patients with and without impaired cerebral autoregulation (ipsilesional VLF phase shift ≤46°) within 7 days.
**Table S3.** Clinical, neuroimaging, cerebral autoregulation characteristics of the patients with and without progressive cognitive decline.
