# Supplementary material for: Impaired cerebral autoregulation is associated with poststroke cognitive impairment
Source: Ann Clin Transl Neurol. 2020 May 28;7(7):1092–102. doi: 10.1002/acn3.51075 (PMC7359112; doi:10.1002/acn3.51075)
Supplement: Supplementary file 1 — Table S1. Comparison of systemic and cerebral hemodynamic parameters between different visits. Table S2. Clinical and neuroimaging characteristics of the patients with and without impaired cerebral autoregulation (ipsilesional VLF phase shift ≤46°) within 7 days. Table S3. Clinical, neuroimaging, cerebral autoregulation characteristics of the patients with and without progressive cognitive decline. [file ACN3-7-1092-s001.docx]

**Supplementary Material**

| **Supplementary Table 1. Comparison of systemic and cerebral hemodynamic parameters between different visits.** | | | | | | | | | | |
| --- | --- | --- | --- | --- | --- | --- | --- | --- | --- | --- |
|  | **Within 7 days** | |  | **3 months** | |  | **1 year** | |  | ***P* value*** |
| **Hemodynamics parameters** | **N** | **Median (IQR)** |  | **N** | **Median (IQR)** |  | **N** | **Median (IQR)** |  |  |
| **Mean blood pressure (mmHg)**† |  |  |  |  |  |  |  |  |  |  |
| **PSCI- (n= 49)** | 49 | 87.8 (76.5 - 95.8) |  | 44 | 81.3 (70.8 - 93.3) |  | 49 | 86.2 (74.2 - 97.2) |  | 0.094 |
| **PSCI+ (n= 16)** | 16 | 79.5 (60.7 - 100.6) |  | 15 | 73.4 (65.1 - 89.4) |  | 13 | 76.4 (68.7 - 83.5) |  | 0.179 |
| **Mean Cerebral blood flow velocity (cm/s)**†‡ |  |  |  |  |  |  |  |  |  |  |
| **PSCI- (n= 49)** |  |  |  |  |  |  |  |  |  |  |
| Ipsilesional | 49 | 32.9 (24.8 - 37.5) |  | 44 | 32.4 (26.7 - 41.0) |  | 49 | 31.4 (26.6 - 36.9) |  | 0.281 |
| Contralesional | 49 | 34.0 (23.8 - 39.4) |  | 44 | 32.4 (26.7 - 46.5) |  | 49 | 32.6 (27.0 - 40.3) |  | 0.102 |
| **PSCI+ (n= 16)** |  |  |  |  |  |  |  |  |  |  |
| Ipsilesional | 16 | 27.5 (21.9 - 33.4) |  | 15 | 33.7 (27.7 - 39.3) |  | 13 | 31.7 (21.3 - 37.1) |  | 0.422 |
| Contralesional | 16 | 30.6 (22.2 -37.4) |  | 15 | 32.0 (24.3 - 39.7) |  | 13 | 29.3 (23.7 - 39.6) |  | 0.885 |
| **Cerebral autoregulation**‡ |  |  |  |  |  |  |  |  |  |  |
| **PSCI- (n= 49)** |  |  |  |  |  |  |  |  |  |  |
| VLF Phase shift (degree) |  |  |  |  |  |  |  |  |  |  |
| Ipsilesional | 49 | 61.9 (46.6 - 81.9) |  | 43 | 54.7 (44.3 - 66.1) |  | 49 | 57.5 (38.8 - 77.9) |  | 0.326 |
| Contralesional | 46 | 60.5 (40.7 - 77.9) |  | 43 | 56.1 (43.9 - 67.4) |  | 47 | 58.0 (44.8 - 75.8) |  | 0.521 |
| VLF Gain (%/%) |  |  |  |  |  |  |  |  |  |  |
| Ipsilesional | 49 | 0.92 (0.75 - 1.42) |  | 43 | 1.16 (0.93 - 1.34) |  | 49 | 1.11 (0.87 - 1.51) |  | 0.300 |
| Contralesional | 46 | 1.05 (0.83 - 1.35) |  | 43 | 1.08 (0.83 - 1.40) |  | 47 | 1.17 (0.89 - 1.60) |  | 0.186 |
| LF Phase shift (degree) |  |  |  |  |  |  |  |  |  |  |
| Ipsilesional | 41 | 39.4 (22.5 - 61.0) |  | 40 | 48.2 (34.5 - 56.8) |  | 40 | 42.5 (20.0 - 57.7) |  | 0.660 |
| Contralesional | 39 | 41.2 (27.3 - 60.1) |  | 41 | 35.5 (23.8 - 52.4) |  | 42 | 47.2 (23.1 - 67.9) |  | 0.148 |
| LF Gain (%/%) |  |  |  |  |  |  |  |  |  |  |
| Ipsilesional | 41 | 1.16 (0.82 - 1.75) |  | 40 | 1.18 (0.89 - 1.95) |  | 41 | 1.50 (1.14 - 1.95) |  | 0.145 |
| Contralesional | 39 | 1.25 (0.95 - 1.70) |  | 41 | 1.12 (0.85 - 1.44) |  | 42 | 1.33 (1.04 - 1.86) |  | 0.088 |
| **PSCI+ (n= 16)** |  |  |  |  |  |  |  |  |  |  |
| VLF Phase shift (degree) |  |  |  |  |  |  |  |  |  |  |
| Ipsilesional | 16 | 41.7 (24.2 - 61.7) |  | 14 | 55.2 (39.1 -69.7) |  | 11 | 41.0 (23.1 - 50.7) |  | 0.121 |
| Contralesional | 13 | 40.0 (28.6 - 47.6) |  | 12 | 52.6 (33.4 - 69.3) |  | 11 | 34.5 (20.4 - 62.9) |  | 0.348 |
| VLF Gain (%/%) |  |  |  |  |  |  |  |  |  |  |
| Ipsilesional | 16 | 1.00 (0.88 - 1.16) |  | 14 | 1.23 (0.66 - 1.66) |  | 11 | 1.05 (0.76 - 1.34) |  | 0.139 |
| Contralesional | 14 | 1.15 (0.96 - 1.88) |  | 12 | 0.93 (0.81 - 1.73) |  | 11 | 1.12 (0.80 - 1.56) |  | 0.806 |
| LF Phase shift (degree) |  |  |  |  |  |  |  |  |  |  |
| Ipsilesional | 12 | 28.8 (18.1 - 52.9) |  | 11 | 36.7 (22.4 - 70.6) |  | 8 | 22.2 (17.7 - 41.6) |  | 0.213 |
| Contralesional | 12 | 25.9 (3.03 - 59.1) |  | 11 | 27.7 (2.4 - 56.8) |  | 9 | 22.6 (8.6 - 45.6) |  | 0.907 |
| LF Gain (%/%) |  |  |  |  |  |  |  |  |  |  |
| Ipsilesional | 12 | 1.13 (0.97 - 1.49) |  | 11 | 1.14 (0.96 - 1.80) |  | 8 | 1.41 (1.19 - 2.73) |  | 0.121 |
| Contralesional | 12 | 1.45 (0.90 - 2.66) |  | 11 | 1.33 (1.17 - 1.69) |  | 9 | 1.17 (0.78 - 2.63) |  | 0.513 |
| *No significant difference was found between different visits on the same side.  †No significant difference was found between patients with and without PSCI at the same visit.  ‡No significant difference was found between bilateral sides at the same visit. | | | | | | | | | | |
| LF, low frequency (0.07 - 0.20 Hz); VLF, very low frequency (0.02 - 0.07 Hz); PSCI, post-stroke cognitive impairment. | | | | | | | | | | |
| The subject numbers of cerebral autoregulation indices might be lower than those of mean cerebral blood flow velocity, which was because the failure of transfer function analysis due to low coherence between mean arterial pressure and mean cerebral blood flow velocity. | | | | | | | | | | |

| **Supplementary Table 2. Clinical and neuroimaging characteristics of the patients with and without impaired cerebral autoregulation (ipsilesional VLF phase shift ≤ 46°) within 7 days** | | | |
| --- | --- | --- | --- |
|  | **Impaired cerebral autoregulation (−) (n = 43)** | **Impaired cerebral autoregulation (+) (n = 22)** | ***P* value** |
| **Age, median (IQR)** | 58 (50 - 64) | 54 (48 - 63) | 0.514 |
| **Male Sex** | 31 (72%) | 17 (77%) | 0.656 |
| **Education level** | 12 (9 - 16) years | 11 (9 - 12) years | 0.046* |
| **Hypertension** | 30 (70%) | 17 (77%) | 0.526 |
| **Mean blood pressure within 7 days, median (IQR)** | 82.8 (71.6 - 94.9) mmHg | 81.3 (76.0 - 111.9) mmHg | 0.792 |
| **Diabetes Mellitus** | 11 (26%) | 11 (50%) | 0.051 |
| **Hemoglobin A1c within 7 days, median (IQR)** | 5.8 (5.4 - 6.2)% | 6.0 (5.6 - 9.3)% | 0.101 |
| **Hyperlipidemia** | 26 (61%) | 20 (91%) | 0.011* |
| **NIHSS score within 7 days, median (IQR)** | 4 (3 - 6) | 3 (1 - 5) | 0.148 |
| **Stroke etiology** |  |  | 0.593 |
| Large artery atherosclerosis | 10 (23%) | 6 (27%) |  |
| Small vessel disease | 30 (70%) | 13 (59%) |  |
| Undetermined etiology | 3 (7%) | 3 (14%) |  |
| **DWI lesion volume, median (IQR)** | 0.7 (0.3 - 2.3) cm^3^ | 0.6 (0.3 - 9.9) cm^3^ | 0.895 |
| **DWI lesion on the left side** | 22 (51%) | 12 (55%) | 0.798 |
| **Fazekas scale score, periventricular + deep white matter, median (IQR)** | 1 (1 - 2) | 1 (0 - 3) | 0.684 |
| **Presence of cerebral microbleeds** |  |  |  |
| Lobar | 13 (30%) | 6 (27%) | 0.805 |
| Deep | 8 (19%) | 7 (32%) | 0.235 |
| Infratentorial | 5 (12%) | 3 (14%) | 0.817 |
| **ICA or MCA stenosis ≥ 70% on either side** | 2 (5%) | 2 (14%) | 0.202 |
| **P*<0.05. | | | |
| DWI, diffusion weighted image; ICA, internal carotid artery; IQR, interquartile range; MCA, middle cerebral artery; MoCA, Montreal Cognitive Assessment; mRS, modified Rankin Scale; NIHSS, National Institute of Health Stroke Scale; PSCI, post-stroke cognitive impairment; VLF, very low frequency (0.02 - 0.07 Hz) | | | |

| **Supplementary Table 3. Clinical, neuroimaging, cerebral autoregulation characteristics of the patients with and without progressive cognitive decline** | | | |
| --- | --- | --- | --- |
|  | **Progressive cognitive decline (−) (n = 53)** | **Progressive cognitive decline (+) (n = 9)** | ***P* value** |
| **Age, median (IQR)** | 57 (50 - 64) | 61 (41 - 66) | 0.787 |
| **Male Sex** | 38 (72%) | 7 (78%) | 0.708 |
| **Education level** | 12 (9 - 14) years | 12 (8 - 13) years | 0.511 |
| **Hypertension** | 40 (76%) | 5 (56%) | 0.219 |
| **Mean blood pressure within 7 days, median (IQR)** | 82.9 (75.8 - 96.4) mmHg | 73.3 (57.5 - 97.8) mmHg | 0.212 |
| **Diabetes Mellitus** | 19 (36%) | 3 (33%) | 0.885 |
| **Hemoglobin A1c within 7 days, median (IQR)** | 5.9 (5.5 - 6.8)% | 5.9 (5.5 - 9.0)% | 0.849 |
| **Hyperlipidemia** | 38 (72%) | 7 (78%) | 0.708 |
| **NIHSS score within 7 days, median (IQR)** | 4 (2 - 5) | 5 (3 - 6) | 0.455 |
| **Stroke etiology** |  |  | 0.385 |
| Large artery atherosclerosis | 13 (25%) | 2 (22%) |  |
| Small vessel disease | 36 (68%) | 5 (56%) |  |
| Undetermined etiology | 4 (8%) | 2 (22%) |  |
| **DWI lesion volume, median (IQR)** | 0.7 (0.3 - 2.8) cm^3^ | 0.5 (0.3 - 3.0) cm^3^ | 0.712 |
| **DWI lesion on the left side** | 26 (49%) | 7 (78%) | 0.113 |
| **Fazekas scale score, periventricular + deep white matter, median (IQR)** | 1 (1 - 2) | 1 (1 - 1) | 0.376 |
| **Presence of cerebral microbleeds** |  |  |  |
| Lobar | 15 (28%) | 2 (22%) | 0.708 |
| Deep | 13 (25%) | 1 (11%) | 0.378 |
| Infratentorial | 7 (13%) | 1 (11%) | 0.863 |
| **ICA or MCA stenosis ≥ 70% on either side** | 4 (8%) | 0 (0%) | 0.398 |
| **Ipsilesional VLF phase shift within 7 days, median (IQR)** | 62° (39° - 78°) | 44° (24° - 61°) | 0.077 |
| **Impaired cerebral autoregulation (ipsilesional VLF Phase shift ≤ 46°) within 7 days** | 16 (30%) | 6 (67%) | 0.036* |
| **P*<0.05. | | | |
| DWI, diffusion weighted image; ICA, internal carotid artery; IQR, interquartile range; MCA, middle cerebral artery; MoCA, Montreal Cognitive Assessment; mRS, modified Rankin Scale; NIHSS, National Institute of Health Stroke Scale; PSCI, post-stroke cognitive impairment. | | | |
